# Simulating ‘that jaw drop moment’: challenging heteronormative assumptions in a novel clinical consultation skills session with undergraduate medical students

**DOI:** 10.15694/mep.2021.000034.2

**Published:** 2021-09-03

**Authors:** Justin Canty, Lynn McBain, Lesley Gray

**Affiliations:** 1 University of Tasmania; 2 University of Otago; 3 University of Otago

**Keywords:** Medical education, consultation skills, sex and gender diversity, simulated patient, curriculum development

## Abstract

This article was migrated. The article was marked as recommended.

Introduction

This paper discusses the design and impact of a clinical consultation skills session for undergraduate medical students in context of diverse sexual orientation, gender identity and expression, and sex characteristics. Existing teaching approaches omit opportunities for application and skills practice. This innovation seeks to address this gap.

Methods

Senior medical undergraduate students participated in actor-facilitated standardized simulated patient role-play. The scenarios utilized a structure akin to the end of year final observed objective structured clinical examination. Plan-do-study-act cycles involving facilitator observation, verbal and written feedback from students and actors, confidential student evaluations, and peer evaluation contributed to session modification and improvement.

Findings

The teaching session offered students the opportunity to practice exam-style simulated patient consultations, communication and empathy skills. Improvements made following the first iteration were reflected in positive student evaluations in the second iteration.

Discussion and Conclusion

Simulated consultations using standardised scenarios represent an accepted format for medical education. We demonstrated it is possible to include topics that frequently give rise to discrimination and stigma from medical professionals whilst maintaining expected learning outcomes. Student evaluations identify the acceptability and value of the topics for medical education. We present a viable option for integration into medical education.

## Introduction

Interpersonal skills, empathy and interactional competencies are increasingly recognized as necessary for effective clinical interactions ( Epstein and Hundert, 2002; Lim *et al*., 2013). This is of particular importance in relation to clinical encounters involving diverse sexual orientation, gender identity, expression, and sex characteristics (SOGIESC). Negative interactions with doctors and other health professionals are routinely identified as a contributing factor to reduced help seeking and poor health outcomes ( Strauss *et al.*, 2017; Koh, Kang and Usherwood, 2014; Pitts *et al.*, 2009; Leonard *et al.*, 2012; Couch *et al.*, 2007).

Assumed heteronormativity and implicit biases relating to sex and gender are part of the broader social landscape in which medical education and medical practice exist ( Ellis, Bailey and McNeil, 2015; Murphy, 2016; Meier and Labuski, 2013). Murphy (2016) argues that medical professionals as members of the wider society are prone to assuming that a patient is heterosexual and cisgender unless informed otherwise. Recent development of Aotearoa New Zealand specific guidelines for gender affirming health care ( Oliphant *et al*., 2018) and broader social change have created a foundation to disrupt such assumptions in medical education and practice. However, being judged, treated poorly, or refused care remain persistent concerns and experiences reported by individuals and communities in research into SOGIESC health ( Strauss *et al*., 2017; Couch *et al.*, 2007; Veale *et al.*, 2019; Riggs, Coleman and Due, 2014). The potent combination of implicit bias and potential for active discrimination contribute to disclosures of diverse sexuality, sex characteristics, or gender identity being experienced as sensitive topics in medical consultations by both patient and doctor. As a result, such disclosures are interactionally fraught and require effective clinical consultation skills to navigate safely. In addition, concern about creating offense or getting a negative reaction from patients by ‘getting it wrong’ can contribute to doctors’ hesitation to ask ( Woodbridge, Dowell and Gray, 2015).

Reviews of medical education curricula internationally and in Aotearoa New Zealand (NZ) identify gaps when it comes to content specific to SOGIESC healthcare and the experiences of people who identify as lesbian, gay, bisexual, transgender, non-binary, and intersex ( Obedin-Maliver *et al*., 2011; DeVita, Bishop and Plankey, 2018; Taylor, Rapsey and Treharne, 2018; Gamble Blakey and Pickering, 2018; Sanchez *et al.*, 2006). Initiatives to redress this balance commonly focus on initial awareness-raising regarding health needs of such communities and exposure to community members’ health care experiences, often through presentations and panel discussions featuring community representatives, such as the intervention for second-year medical students developed by Kelley *et al*., (2008). A critical gap in such approaches is the lack of opportunity for students to practice consultation skills with a specific focus on SOGIESC diverse people in relation to their health, in relation to either routine or specific health needs.

This gap has been identified in evaluation feedback on teaching sessions that have had a solely awareness raising focus ( Kelley *et al.*, 2008; Hanssmann, Morrison and Russian, 2008). In a study of medical students’ confidence for working with SOGIESC populations, Sanchez *et al*., (2006) found that students with increased clinical exposure to people with sex and gender diversity (SGD) tended to perform more comprehensive histories, held more positive attitudes and greater knowledge of SGD health care concerns than those who had little or no clinical exposure. These key insights into gaps and learning processes provided the pedagogical foundation for developing the clinical consultation skills session discussed here, with structure and timing similar to the observed objective structured clinical examination (OSCE). The OSCE, first described by Harden and colleagues in the 1970’s ( Harden *et al.*, 1975) continues as a familiar component of medical assessment around the world in undergraduate and postgraduate clerkships, fellowship examinations and registration to practice medicine ( Tervo *et al.*, 1997; Medical Council of New Zealand, n.d.; Nuovo, Bertakis and Azari, 2006). Significant for the focus of this discussion, Hodges (2003) poses a question regarding the place of diversity (e.g. gender, race, culture, religion) in what is a homogenized process. The OSCE is included in teaching and assessment of communication skills and increasingly as a vehicle for diverse and challenging topics including cultural competence, palliative care and alcohol or other drugs counselling ( Brazeau, Boyd and Crosson, 2002; Yedidia *et al.*, 2003; Deveugele *et al*., 2005; Rider, Hinrichs and Lown, 2006; Miller and Green, 2007; Sloan *et al*., 2001; Stein, Parish and Arnsten, 2005; Parish *et al*., 2006).

This article discusses an improvement cycle undertaken on a SOGIESC focused teaching session and scenarios. The innovative approach using simulated clinical consultation skills practice to sensitize undergraduate medical students to the relevance of SOGIESC health for primary health care and general practice is described.

## Method

### Subjects

Two groups of year five (penultimate year in NZ) medical students in 2016. Each comprised approximately 30 students. These students were due to undertake summative Objective Structured Clinical Examinations (OSCE) at the end of the academic year.

### Taboo Topics Teaching Session

All sessions were scheduled during the Primary Health Care and General Practice block teaching module and as such were integrated into the regular teaching and learning activities during this rotation. The sessions involved actor-facilitated standardized simulated patient role play activities, using a structure and a 7-minute time limit, akin to the upcoming summative OSCE assessment conditions. These were conducted in small groups in a ‘fishbowl’ arrangement, with students who were not engaged in the doctor role observing the interaction. Students received feedback from peers, actors, and teachers. Session planning allowed for 3-4 iterations, dependent on the time available for the whole session.

These simulations were followed by general feedback on consultation skills to the whole group from the actors, and then followed by seminar style lesson focused on specific content linked to vocabulary and skills for engaging with patients around SOGIESC experiences.

Students were provided with brief information about the patient prior to the simulation. Students worked in groups of approximately 6-7 students, each group completing two or three different scenarios with simulated actors rotating between groups. Two teachers (LG, JC) circulated between groups to support actors and students when specific queries emerged. This additional support was provided in recognition of the anticipated and likely low prior exposure to SOGIESC specific content knowledge ( Taylor, Rapsey and Treharne, 2018). One teacher was also responsible for keeping time (LG).

### Scenarios

Standardized simulated patient scenarios were developed where disclosure of ‘sensitive’ information potentially impacted on the presenting issue, to the doctor-patient relationship, or both. They include situations where the person’s physical attributes were salient to differential diagnosis or treatment planning (e.g. transgender man with UTI), where a personal attribute potentially influenced the doctor-patient relationship (e.g. lesbian woman presenting with abdominal pain angry about past doctors asking if she might be pregnant), and ‘breaking bad news’ scenarios (e.g. patient with complete androgen insensitivity syndrome seeking testosterone therapy for masculinizing). Most scenarios were developed from genuine GP consultations recounted to the authors, then de-identified and used with the permission of the ‘tellers’. Some additional scenarios were developed as composite patients from discussions with community worker colleagues and published material ( O’Donnell and Taylor, 2014; Intersex Human Rights Australia Ltd n.d.).

The actor is provided with a full outline of the simulated patient ‘story’ including background and simulated patient cues, near patient test findings, thoughts and feelings connected with the consultation, and presenting issue [session outline and scenario scripts are available on request to the corresponding author].

### Quality Improvement Tools

To inform the planned quality improvement we followed a plan-do-study-act (PDSA) cycle approach ( F., R., 1940), a common feature of health service quality improvement ( NHS Improvement, 2018) and medical education learning theory ( Cleghorn and Hendrick, 1996). The PDSA cycle is useful for iterative cycles of quality improvement ( Cleghorn and Hendrick, 1996). Academic peer review of the session was obtained from a trusted professional colleague with SOGIESC content knowledge. JC and LG critically appraised the first iteration from the perspective of teacher and facilitator. Verbal and written feedback was obtained from students and simulated patient actors. Confidential student evaluations of teaching were collected independently following each session through the University of Otago’s Quality Advancement Unit (QAU). The survey comprises a combination of four questions on a five point Likert-type scale and three questions with free text response boxes.

For the iterations of the session discussed below, the scenarios remained unchanged. Aspects of the session outline, actor briefing, and time allocations were adjusted in response to feedback from these quality improvement processes.

## Results

### Participants

Fifty-eight fifth year medical students (29 students in each module group), participated in the sessions. Teachers (JC and LG) received unprompted verbal feedback from students during and immediately after each session.

The confidential online survey invitation link was sent direct to all students via email shortly after each session. The survey was open for two weeks, with a reminder email after one week. Seven students from group one (G1) responded to the survey (7/29, 24% of class) and 24 students from group two (G2) responded (24/29, 83% of class). The low student response rate from G1 coincided with a University move from paper-based evaluations (completed in class under the direction of an administrator) to online format only.

### Scenarios as learning tools

Students in this study highly rated the activities as engaging to a large or very large extent (G1 71% ; G2 79%). The scenarios were not changed for S2 for students, although feedback from the simulated patient actors indicated more clarity and background details were required for the actors in preparation for G2. These high ratings were supported by written feedback from students when asked “what helped your learning in this session?”, and peer reviewer observation -

*It was good to have some practical patient consultation scenarios to work on* (G1, student 1)

*OSCE practise w/ real scenarios was very valuable* (G2, student 2)

*Provides relevance to the students and presents them with situations they may not have thought of* (peer reviewer).

### That ‘jaw drop moment’

The scenarios occur early in the session with no prior preparation or teaching on the topic focus. JC and LG had felt there were benefits to placing the scenarios earlier in the session to expose the students to consultations where they have no idea who is coming through the door or what the presenting issue and personal history might be, as is the case in many general practice consultations. As facilitators, JC and LG observed many ‘jaw drop moments’ in G1 and G2 when the students realized elements of the scenario were unfolding in a way they had not expected or planned for. Many of the students commented on this as a positive, verbally and in writing. A small number of students in G1 commented that they would have preferred some prior warning of the cases so that they could prepare although the small number of G1 students responding to the online survey (n=7) did not explicitly mention this as an issue. Responses from G1 students were largely positive -

*I really enjoyed the advice on communicating and consulting with LGBTIQA patients “how do you identify?” “What does that mean for you?” etc. The advice was practical and easy to follow. I hope to put it to use in the future* (G1, student 1)

The peer reviewer suggested -

perhaps have a minute or two more introduction as to why when you are doing the outline, I think you briefly talked re clinical skills, maybe just a bit more on context but not so much it gives away what you are hoping to occur in the OSCE role-play (peer reviewer).

The teachers strengthened the introduction regarding communication skills to successfully and safely negotiate a consultation with minimal or no prior case knowledge. The ‘jaw drop moment’ scenarios proceeded as originally planned. Written comments were captured from S2 students -

*Dealing with difficult situations in the OSCEs were great, also being put on the spot was good* (S2, student 13)

*Scenarios made you think about how to phrase difficult questions* (S2, student 14)

### Teaching dynamics

The teaching team of JC and LG worked well together and this was evident in the feedback received -

*The tutorial leaders were really good! Very honest, open environment* (S2, student 4)

*Really well-run balanced session. Very helpful!* (S2, student 18)

*Both the tutors were great & engaging = great to practice some OSCEs* (S2, student 21)

### Session format challenges

Session timing presented a challenge to incorporate knowledge and skills content. Three hours were allocated during a two-week module with no opportunity for other or additional time or resources to fund additional simulated patient actors. For resource (staff/actors) and time efficiency all the student groups were in the same large lecture room at breakout tables. In G1, groups for scenario activities were large and this inhibited interaction for some students -

*Sometimes I didn’t feel comfortable discussing and asking questions in the group setting* (G1, student 3)

We increased the number of simulated patient actors to facilitate a larger number of smaller group sizes in G2. Having more actors and a larger number of breakout groups in the same larger lecture room brought its own challenges -

*Having multiple OSCEs in one room was distracting at times* (S2, student 6)

Although many students embraced the adjusted structure -

*The break up of all activities & involvement really helpful. Enjoyed openness & OSCE situations to practise* (S2, student 11)

*A well structured session with a good balance of interactive OSCE work & group learning* (S2, student 12)

*Really well-run balanced session. Very helpful!* (S2, student 24)

While the number of G1 survey respondents were small (7; 24%), there was a notable difference in median responses in G2 across all LTS responses with LTS 1 being the highest and LTS 5 the lowest. The session was rated highly for improving student understanding of concepts relating to the topic (G1 median 2.2; G2 median 1.8). The largest differences were in relation to ability to communicate this subject matter (G1 median 2.7; G2 median 1.8) and the ability to develop competency in this area (G1 median 2.8; G2 median 1.8) and this is likely to have been related to the limited number of scenario opportunities in S1 and the associated increase in engagement in G2 (G1 median 2.1; G2 median 1.5). This suggests the adjustments made to G2 positively enhanced the overall learning experience.

## Discussion

Scenarios with simulated patient actors established an effective ‘buy in’ to the session on non-assessable content. This practice opportunity using comparable examination conditions in time frame, actor-supported simulations, and feedback from peers and tutors created grounds for students’ active engagement in the sessions. The overall structure of G2 received a higher positive response rate to the LTS evaluation questions compared to G1. We believe this highlighted the perceived value of a repeated opportunity to practice consultation skills using standardised patient scenarios akin to students’ upcoming OSCE assessment. This projected engagement strategy was frequently nominated by student comments as a valuable dimension of the session.

Consultation skills teaching routinely introduces the topic and intended learning outcomes of the session or module in preparatory materials and introductory exposition of the session, consistent with quality university teaching practice ( Biggs and Tang, 2011). This alerts students to the skill focus of the instruction. However, it diminishes much of the potential for introducing unpredictability even where it is linked to the skill focus. We wanted to ascertain whether the sequence change described above was a viable means to simulate the ‘surprise’ aspect of primary care consultations, combined with the interactional ‘work’ involved with talk about sensitive personal topics. We also sought to build on this simulated experience of unanticipated disclosures from patients to explore and challenge implicit bias in a safe educational environment with trained actors. The moment of realization or disclosure of a patient’s experience as different from assumed heteronormativity is what we refer to in this article as the ‘jaw drop’ moment. Students had an expected norm of explanation prior to practice and some students voiced concerns relating to this. Being ‘put on the spot’ or having to manage an unexpected turn in a consultation was frequently described as a positive learning experience. The majority of students were observed responding favourably in session, knowing this was a space for practice and (relative) safety to make mistakes prior to encountering real-world patients. This further demonstrated to us that the structure of the session and the design of the scenarios were effective in simulating teachable opportunities.

Challenging implicit biases has become an important step to redress their effects within the medical curriculum and subsequent medical practice ( Fallin-Bennett, 2015). Situating this challenge within a session focused on empathy in consultation skills offers an important micro level setting to improve equity for marginalized populations ( Sukhera, 2019). Implicit biases need to be illuminated in a way that students can become sensitized to their existence and effects that in turn create an impact on their approach to practice. Being situated in a primary health care and general practice department enabled us to consider the qualities of primary care consultations where consultations are characteristically unpredictable, and the doctor frequently receives no prior alert to the reason for patient attending. This ‘element of surprise’ characteristic of primary care consultations offered a unique and valuable pedagogical context to illuminate and challenge students on the effects of implicit bias influencing their perceptions and approach.

It has previously been reported that time dedicated to sex and gender related topics to be very small with variability in quality of teaching ( Obedin-Maliver *et al.*, 2011). Similarly, Gamble Blakey and Treharne (2019) highlight challenges in advancing transgender healthcare teaching in NZ. An already ‘full’ curriculum is recognized as an issue in the design of undergraduate medical education in NZ, where addition of content is generally paired with loss of other content ( Taylor, Rapsey and Treherne, 2018). However, ‘addition’ is not the only option. Integration rather than addition offers another solution and also advocated by Obedin-Maliver *et al.*, (2011). This session presents a viable option for integration of SOGIESC topics into medical education and there is an appetite for such resources as evidenced by the requests received for the scenarios following an oral presentation relating to teaching skills at the Australian and New Zealand Professional Association for Transgender Health ( Canty and Gray, 2017).

## Strengths and Limitations

There were consistent comments in student evaluations wanting more teaching in this area, including knowledge input as well as further practice of simulated consultations. We acknowledged to students that it was impossible to cover all the relevant knowledge to SOGIESC health in the session and provided a focused selection of resources for students to use for further study. This needs to be supported elsewhere across the curriculum.

Some scenarios were challenging for students to manage in the absence of content knowledge. This commonly emerged in relation to intersex condition scenarios but also affected sexuality and transgender focused scenarios.

There was a low student response rate to the online questionnaire following G1. This coincided with a University move from paper-based evaluations completed in class under the direction of an administrator to online format only and in initial implementation low response rates were noted as commonplace amongst teaching staff.

## Conclusion

Marginalization within medical education replicates the marginalization of SOGIESC populations in the wider society, which in turn contributes to health inequities rather than reducing them. Thus, it is vital for medical education to integrate SOGIESC diversity into the curriculum as a step towards reducing health inequities for these populations. This novel and innovative session demonstrated the potential for integrating a practice skills focus based on empathy and rapport skills using simulated consultations as a viable and effective pedagogical approach to integrating clinical skills practice specifically related to SOGIESC in medical education. This was achievable in a single session, placing consultation practice skills at the center, a dimension that was absent in other approaches. Providing opportunities for students to practice in safe simulated scenarios offers a vital development for incorporating diverse sexualities, sex, and gender health care into undergraduate medical education. Research is needed to assess the impact of the session content on heteronormative assumptions and implicit biases of the students and if and how this session influences their practice going forward.

## Take Home Messages

• Increasing medical student sensitisation to challenges in disclosing sexual orientation, gender identity, gender expression, and sex characteristics (SOGIESC) is a significant and desired inclusion in the curriculum.
• Existing approaches to SOGIESC teaching often focus on increasing awareness but omit opportunities for skills practice.
• Simulated consultations using standardised patient scenarios represent an accepted format for achieving such sensitisation in undergraduate medical education, achievable in a single stand-alone session.
• Students highly value safe and supported sessions simulating non-binary disclosures and wanted more SOGIESC health teaching.
• Consultation skills for responding to SOGIESC health needs require support from integrated content in medical education curriculum.

## Notes On Contributors

Justin Canty*, PhD, BSW, Grad Dip MH, PG Cert Health Sci (CAMH), Lecturer in Social Work, University of Tasmania. Justin’s research and teaching focuses on interactional analysis of social work encounters and social work practice skills. His current project investigates empathy in social work encounters with trans and gender diverse people.

**Lynn McBain** *,*BSc, MD, FRNZCGP(Dist) is Associate Professorand HOD of the Department of Primary Health Care and General Practice, University of Otago Wellington. Her research interests include delivery of innovation in medical education, quality in prescribing and health systems evaluation.

Lesley Gray*, MPH, MSc, FFPH, PGCertEd, FETC, is Senior Lecturer with the Department of Primary Health Care and General Practice, University of Otago, Wellington, teaching years 3-6 of the MBChB New Zealand medical degree. Lesley’s research interests centre on risk communication, risk reduction, discrimination and bias pertaining to health equity for marginalised populations. ORCID ID: http://www.orcid.org/0000-0001-6414-3236

^*^Co-first authors - these authors contributed equally to this work.

## References

[ref1] Biggs J. and Tang C. (2011) Teaching for quality learning at university. 4thed. Maidenhead, Berkshire, England: Open University Press/McGraw-Hill.

[ref2] Brazeau C. Boyd L. and Crosson J. (2002) Changing an existing OSCE to a teaching tool: the making of a teaching OSCE. Acad Med. 2002;77:932. 10.1097/00001888-200209000-00036 12228103

[ref3] Canty J. and Gray L. (2017) The Last taboo? Teaching skills for clinical consultations with sex/gender diverse people in medical education.In 4 ^th^ Australian and New Zealand Professional Association for Transgender Health Biennial Conference. Sydney, Australia.

[ref4] Cleghorn G. D. and Headrick L. A. (1996) The PDSA cycle at the core of learning in health professions education. The Joint Commission Journal on Quality Improvement. 22(3), pp.206–12. 10.1016/S1070-3241(16)30223-1 8664953

[ref5] Couch M. Pitts M. Mulcare H. Croy S. (2007) TranZnation: A report on the health and wellbeing of transgendered people in Australia and New Zealand. Melbourne: Australian Research Centre in Sex, Health & Society, La Trobe University. Available at: https://apo.org.au/node/3849( Accessed: 21 January 2021).

[ref6] Deveugele M. Derese A. De Maesschalck S. Willems S. (2005) Teaching communication skills to medical students a challenge in the curriculum? Patient Education and Counseling. 58, pp.265–270. 10.1016/j.pec.2005.06.004 16023822

[ref7] DeVita T. Bishop C. and Plankey M. (2018) Queering medical education: systematically assessing LGBTQI health competency and implementing reform. Medical Education Online. 23(1), pp.1510703. 10.1080/10872981.2018.1510703 30157712 PMC6116674

[ref8] Ellis S. J. Bailey L. and McNeil J. (2015) Trans people’s experiences of mental health and gender identity services: A UK study. Journal of Gay & Lesbian Mental Health. 19(1), pp.4–20. 10.1080/19359705.2014.960990

[ref9] Epstein R. M. and Hundert E. M. (2002) Defining and assessing professional competence. JAMA. 287(2), pp.226–35. 10.1001/jama.287.2.226 11779266

[ref10] F. R. (1940) Statistical method from the viewpoint of quality control. Nature. 146, pp.150. 10.1038/146150e0

[ref11] Fallin-Bennett K. (2015) Implicit bias against sexual minorities in medicine: cycles of professional influence and the role of the hidden curriculum. Academic Medicine. 90(5), pp.549–52. 10.1097/acm.0000000000000662 25674911

[ref12] Gamble Blakey A. G. and Treharne G. J. (2019) Advancing transgender healthcare teaching in Aotearoa/New Zealand. The New Zealand Medical Journal (Online). 132(1491), pp.104–6. 30845137

[ref13] Gamble Blakey A. and Pickering N. (2018) Putting it on the table: towards better cultivating medical student values. Medical Science Educator. 28(3):533–42. 10.1007/s40670-018-0584-8

[ref14] Hanssmann C. Morrison D. and Russian E. (2008) Talking, gawking, or getting it done: Provider trainings to increase cultural and clinical competence for transgender and gender-nonconforming patients and clients. Sexuality Research & Social Policy. 5(1), pp.5–23. 10.1525/srsp.2008.5.1.5

[ref15] Harden R. T. M. Stevenson M. Downie W. W. and Wilson G. M. (1975) Assessment of clinical competence using objective structured examination. British Medical Journal. 1(5955), pp447–451. 10.1136/bmj.1.5955.447 1115966 PMC1672423

[ref16] Hodges B. (2003) OSCE! Variations on a theme by Harden. Med Educ. 37(12), pp.1134–40. 10.1111/j.1365-2923.2003.01717.x 14984124

[ref17] Intersex Human Rights Australia Ltd. (n.d.). Personal stories. Available at: https://ihra.org.au/category/comment/personal-stories/( Accessed: 16 September 2020).

[ref18] Kelley L. Chou C. L. Dibble S. L. and Robertson P. A. (2008) A critical intervention in lesbian, gay, bisexual, and transgender health: knowledge and attitude outcomes among second-year medical students. Teaching and Learning in Medicine. 20(3), pp.248–53. 10.1080/10401330802199567 18615300

[ref19] Koh C. S. Kang M. and Usherwood T. (2014) ‘I demand to be treated as the person I am’: experiences of accessing primary health care for Australian adults who identify as gay, lesbian, bisexual, transgender or queer. Sex Health. 11(3), pp.258–64. 10.1071/SH14007 24990299

[ref20] Leonard W. Pitts M. Mitchell A. Lyons A. (2012) Private lives 2. The second national survey on the health and wellbeing of gay, lesbian, bisexual, transgender (GLBT) Australians. Melbourne: The Australian Research Centre in Sex Health and Society, La Trobe University.

[ref21] Lim. B. T. Moriarty H. Huthwaite M. Gray L. (2013) How well do medical students rate and communicate clinical empathy? Medical Teacher. 1;35(2), pp.e946–51. 10.3109/0142159X.2012.715783 22938688

[ref22] Medical Council of New Zealand. (n/d) Registration exam. Available from: https://www.mcnz.org.nz/registration/getting-registered/registration-exam-nzrex/( Accessed: 5 January 2020).

[ref23] Meier S. C. Labuski C. M. (2013) The demographics of the transgender population.in Baumle A. K. (ed) International handbook on the demography of sexuality. Dordrecht: Springer, pp.289–327.

[ref24] Miller E. and Green A. R. (2007) Student reflections on learning cross-cultural skills through a ‘cultural competence’ OSCE. Medical Teacher. 29(4), pp.e76–e84. 10.1080/01421590701266701 17786736

[ref25] Murphy M. (2016) Hiding in plain sight: The production of heteronormativity in medical education. Journal of Contemporary Ethnography. 45(3), pp.256–89. 10.1177/0891241614556345

[ref26] NHS Improvement. (2018) Plan, Do, Study, Act (PDSA) cycles and the model for improvement. Available at: https://improvement.nhs.uk/resources/pdsa-cycles/( Accessed: 16 September 2020).

[ref27] Nuovo J. Bertakis K. D. and Azari R. (2006) Assessing resident’s knowledge and communication skills using four different evaluation tools. Medical Education. 40, pp.630–636. 10.1111/j.1365-2929.2006.02506.x 16836535

[ref28] Obedin-Maliver J. Goldsmith E. S. Stewart L. White W. (2011) Lesbian, gay, bisexual, and transgender–related content in undergraduate medical education. JAMA. 306(9), pp.971–7. 10.1001/jama.2011.1255 21900137

[ref29] O’Donnell M. and Taylor B. (2014) Working Therapeutically with LGBTI Clients; A Practice Wisdom Resource. Newtown NSW: LGBTIQ+ Health Australia. Available at: https://d3n8a8pro7vhmx.cloudfront.net/lgbtihealth/pages/660/attachments/original/1585379519/Practice-Wisdom-Guide-ONLINE.pdf?1585379519( Accessed: 21 January 2021).

[ref30] Oliphant J. Veale J. Macdonald J. Carroll R. (2018) Guidelines for gender affirming healthcare for gender diverse and transgender children, young people and adults in Aotearoa, New Zealand. Transgender Health Research Lab, University of Waikato, 2018, ( Accessed: 16 October 2020). Available at: https://researchcommons.waikato.ac.nz/bitstream/handle/10289/12160/Guidelines%20for%20Gender%20Affirming%20Health 30543615

[ref31] Parameshwaran V. Cockbain B. C. Hillyard M. and Price J. R. (2017) Is the lack of specific lesbian, gay, bisexual, transgender and queer/questioning (LGBTQ) health care education in medical school a cause for concern? Evidence from a survey of knowledge and practice among UK medical students. Journal of Homosexuality. 64(3), pp.367–81. 10.1080/00918369.2016.1190218 27184023

[ref32] Parish S. J. Ramaswamy M. Stein M. R. Kachur E. K. (2006) Teaching about substance abuse with Objective Structured Clinical Exams. Journal of General Internal Medicine. 21(5), pp.453–459. 10.1111/j.1525-1497.2006.00426.x 16704387 PMC1484780

[ref33] Pitts M. K. Couch M. Mulcare H. Croy S. (2009) Transgender people in Australia and New Zealand: Health, well-being and access to health services. Feminism & Psychology. 19(4), pp.475–95. 10.1177/0959353509342771

[ref34] Rider E. A. Hinrichs M. M. and Lown B. A. (2006) A model for communication skills assessment across the undergraduate curriculum. Medical Teacher. 28(5), pp.e127–e134. 10.1080/01421590600726540 16973446

[ref35] Riggs D. W. Coleman K. and Due C. (2014) Healthcare experiences of gender diverse Australians: a mixed-methods, self-report survey. BMC Public Health. 14(1), pp.230. 10.1186/1471-2458-14-230 24597614 PMC3973980

[ref36] Sanchez N. F. Rabatin J. Sanchez J. P. Hubbard S. (2006). Medical students’ ability to care for lesbian, gay, bisexual, and transgendered patients. Family Medicine. 38(1), pp.21–27. 16378255

[ref37] Sloan P. A. Plymale M. A. Johnson M. Vanderveer B. (2001) Cancer pain management skills among medical students: the development of a Cancer Pain Objective Structured Clinical Examination. Journal of Pain and Symptom Management. 21, pp.298–306. 10.1016/S0885-3924(00)00278-5 11312044

[ref38] Stein M. R. Parish S. J. and Arnsten J. H. (2005) The OSCE as a formative evaluation tool for substance abuse teaching. Medical Education. 39(5), pp.529–530. 10.1111/j.1365-2929.2005.02147.x 15842715 PMC2692590

[ref39] Strauss P. Cook A. Winter S. Watson V. (2017) Trans Pathways: the mental health experiences and care pathways of trans young people. Summary of results. Telethon Kids Institute, Perth, Australia. Available at: https://www.telethonkids.org.au/globalassets/media/documents/brain--behaviour/trans-pathways_plain-text_no-figures.pdf( Accessed: 16 October 2020).

[ref40] Sukhera J. (2019) Empathy and Implicit Bias: Can Empathy Training Improve Equity?In: Teaching Empathy in Healthcare. Springer: Cham, pp.223–238.

[ref41] Taylor O. Rapsey C. and Treharne G. (2018) Sexuality and gender identity teaching within preclinical medical training in New Zealand: content, attitudes and barriers. New Zealand Medical Journal. 131(1477), pp.35–44. 29927914

[ref42] Tervo R. C. Dimitrievich E. Trujillo A. L. Whittle K. (1997) The Objective Structured Clinical Examination (OSCE) in the clinical clerkship: an overview. South Dakota J Med. 50, pp.153–156. 9155233

[ref43] Veale J. Byrne J. Tan K. Guy S. (2019) Counting Ourselves: The health and wellbeing of trans and non-binary people in Aotearoa New Zealand. Transgender Health Research Lab, University of Waikato: Hamilton NZ. Available at: https://countingourselves.nz/wp-content/uploads/2019/09/Counting-Ourselves_FINAL.pdf( Accessed: 17 September 2020).

[ref44] Woodbridge M. Dowell A. and Gray L. (2015) “He said he had been out doing the traffic” General Practitioner perceptions of STI and HIV testing strategies for men. J Prim Health Care. 7(1), pp.50–56. 10.1071/hc15050 25770716

[ref45] Yedidia M. J. Gillespie C. Kachur E. Schwartz M. D. (2003) Effect of communications training on medical student performance. J Am Med Assoc. 290, pp.1210–1212. 10.1001/jama.290.9.1157 12952997

