# Supplementary material for: Simulating ‘that jaw drop moment’: challenging heteronormative assumptions in a novel clinical consultation skills session with undergraduate medical students
Source: MedEdPublish (2016). 2021 Sep 3;10:34. Originally published 2021 Feb 4. [Version 2] doi: 10.15694/mep.2021.000034.2 (PMC10939589; doi:10.15694/mep.2021.000034.2)
Supplement: Peer review history

# Reviewer report: Reviewer response for version 2

**DOI:** 10.21956/mep.20210.r31290

**Published:** 2021-09-18

**Authors:** Ken Masters

**Affiliations:** 1 Sultan Qaboos University

This review has been migrated. The reviewer awarded 4 stars out of 5 Although the authors have made small changes to the paper, it is a pity that the new version has not reflected the full potential of what was done in the study. Because of the importance of the topic, the paper is still a worthwhile read, but I really am left with a sense of absence of what could have been accomplished by their study if they had presented their work as formal research. This would have been an ideal opportunity to create an almost seminal paper on the topic that would have been essential reading for anyone wishing to introduce the topic into their courses. I look forward to seeing formal research on this topic.

Reviewer Expertise:

NA

No decision status is available

# Reviewer report: Reviewer response for version 2

**DOI:** 10.21956/mep.20210.r31291

**Published:** 2021-09-07

**Authors:** Leila Niemi-Murola

**Affiliations:** 1 University of Helsinki

This review has been migrated. The reviewer awarded 4 stars out of 5 This is an interesting article focusing on challenging heteronormative assumptions. The perspective is novel for me and I started to reflect my own thoughts when reading it. We as healthcare workers have stopped taking for granted than the mother’s husband would be the father of her child, and one can see rainbow signs and flags everywhere. However, our patients will have to correct our assumption that they live heteronormative lives. The authors have included a thorough review of the literature in the article, which helps the reader to see the gap between our communication skills curriculum and the reality in which some of our patients live in. The authors have an innovative curriculum to give tools for the students to encounter SOGIESC patients and also tools for teachers for implementing this in their own curriculum.

Reviewer Expertise:

NA

No decision status is available

# Reviewer report: Reviewer response for version 1

**DOI:** 10.21956/mep.19016.r27292

**Published:** 2021-05-12

**Authors:** Ken Masters

**Affiliations:** 1 Sultan Qaboos University

This review has been migrated. The reviewer awarded 3 stars out of 5 This is an interesting paper on the introduction of sexual orientation and related aspects and characteristics of patients into an OSCE.The topic is certainly one that needs to be addressed, and I look forward to a day when these issues are no longer “jaw-dropping”, but are simply part of the anticipated possibilities.It is a pity, though, that the researchers chose to perform this work in such an informal manner. Having obtained ethics approval, and having all the subjects, it would have been far more valuable if it had been conducted as formal research and then the data published as such (from the description of what was done, it appears that it MAY have been formally conducted, but more details are required and the Results presentation does not reflect this). The paper would have fitted far more easily into the Research category. (It does not sit comfortably into the category of “New education method or tool”, because simulations and OSCEs have long been used to teach and assess clinical skills within a context that challenges social norms and expectations (e.g. different cultures, races, abilities, etc). The novelty of this paper is not the method or the tool, but rather the topic, and the authors have missed an opportunity to present their findings as solid research.)I’m not quite sure what can be done about this. If the authors still have all their original data, then I would recommend that they:• Present their quantitative data formally, and• Formally theme their qualitative data and present these also in their themes.The “Methods” section would have to include a description of these processes.If they do not wish to do this, then I hope they will conduct further research on the topic.So, while the paper is a useful alert, and idea for others to try, it would be more valuable to the readers if the work were presented more formally as research.

Reviewer Expertise:

NA

No decision status is available

# Reviewer report: Reviewer response for version 1

**DOI:** 10.21956/mep.19016.r27291

**Published:** 2021-02-08

**Authors:** Leila Niemi-Murola

**Affiliations:** 1 University of Helsinki

This review has been migrated. The reviewer awarded 3 stars out of 5 This is an interesting article focusing on design of a simulated consultation skills session for medical undergraduate students. Only recently there has been public discussion about health care personnel and their encounter with patients having diverse sexual orientation. As the authors state, heteronormative orientation is often taken as the normal, and physicians have scarce tools to adequately cope with patients not having this kind of orientation. However, the authors do not report asking the students’ perceptions prior to the study.The students participating the study were due to undertake an OSCE. It is a bit unclear if the study was a part of the OSCE or an OSCE-style session preparing the students for an examination. It is not clear either if participation was voluntary and if the students gave their informed consent before the study. Obviously, it was voluntary to take the survey. In the results section the authors reported that feedback was collected between the groups but the scenarios were not changed. This should have been mentioned in the methods section.The idea of the study is very attractive but it was a bit challenging to understand design and setting of the study.

Reviewer Expertise:

NA

No decision status is available

# Reviewer report: Reviewer response for version 1

**DOI:** 10.21956/mep.19016.r27293

**Published:** 2021-02-04

**Authors:** Ashleigh Rushton

This review has been migrated. The reviewer awarded 5 stars out of 5 Very informative piece that showcases the value of integrating discussions and learning opportunities for medical students in relation to SOGIESC within medical education.

Reviewer Expertise:

NA

No decision status is available
